# Expression of Concern

**DOI:** 10.1080/19336950.2025.2570092

**Published:** 2025-10-09

We, the Publisher and Editor of *Channels* are issuing an Expression of Concern for the following article:

**Liu, J., Supnet, C., Sun, S., Zhang, H., Good, L., Popugaeva, E., & Bezprozvanny, I. (2014). The role of ryanodine receptor type 3 in a mouse model of Alzheimer disease. *Channels, 8*(3), 230–242**. **https://doi.org/10.4161/chan.27471**

Following publication, concerns were raised by a third party in 2025 regarding the integrity of the data shown in Figure 4. Investigations conducted by the Journal and Publisher confirm that there are unexpected similarities between images representative of different samples in Figure 4A. Whilst the authors have responded, given the length of time since publication, they have been unable to provide the original data, and so, the concerns remain unaddressed.

The journal wishes to advise that the concerns raised do not affect the overall findings of the article, and there are no other integrity concerns with the article.

Thus, no further action will be taken at this time, however, we advise readers to interpret the information presented in Figure 4A with due caution.

The authors have been notified about this Expression of Concern. Should further information come to light, the journal will reassess any concerns raised.

